# SYSCILIA, “A systems biology approach to dissect cilia function and its disruption in human genetic disease”

**DOI:** 10.1186/2046-2530-1-S1-P41

**Published:** 2012-11-16

**Authors:** R Roepman, M Ueffing, H Kremer, MA Huynen, TJ Gibson, N Katsanis, GT Walz, U Wolfrum, B Franco, RH Giles, PL Beales, CA Johnson, OE Blacque, M Pontoglio, F Képès, G Apic, RB Russell, H Omran

**Affiliations:** 1 Nijmegen Centre for Molecular Life Sciences, Radboud University Nijmegen Medical Centre, the Netherlands; 2 Medical Proteome Center, Center of Ophthalmology, University of Tuebingen, Germany; 3 European Molecular Biology Laboratory, Heidelberg, Germany; 4 Duke University Medical Cente, USA; 5 Universitätsklinikum Freiburg, Germany; 6 Johannes Gutenberg Universitaet Mainz, Germany; 7 Telethon Institute of Genetics and Medicine, Naples. Italy; 8 Universitair Medisch Centrum Utrecht, the Netherlands; 9 University College London, UK; 10 The Leeds Institute of Molecular Medicine, UK; 11 University College Dublin, Ireland; 12 Institut National de la Sante et de la Recherche Medicale, France; 13 Centre National de la Recherche Scientifique, France; 14 Cambridge Cell Networks Ltd., UK; 15 Ruprecht-Karls Universitaet Heidelberg, Germany; 16 Westfaelische Wilhelms Universitaet Muenster, Germany

## 

Primary cilia are basically signaling hubs, harboring amongst others the noncanonical WNT, Hedgehog,and PDGF signaling systems, and their disruption leads to striking developmental defects. Some ciliopathy-associated proteins have recently been revealed to be physically or functionally associated in several distinct groupings, with limited connections to other crucial biological processes. Early proteomics studies have also suggested a discrete repertoire of about 1000 proteins within the organelle (i.e. <5% of the proteome) that are still in need of organisation into pathways and networks. Small, relatively isolated systems are often targeted by systems biology approaches under the assumption that a limited set of molecules and interactions will be more tractable for modelling systems. Cilia are thus ideal organelles for systems biology as they can be regarded as semi-closed systems being both largely spatially and biologically separated from many other cellular structures and processes.

## Scientific and technical objectives

Our overall objectives are to establish a paradigm for studying and modelling complex eukaryotic systems, to understand how system perturbation contributes to the modulation of clinical phenotypes, and to provide a better understanding of ciliary processes in biology and their associated diseases. Our objectives focus on all critical components of the systems biology process, namely:

• assay development and application

• data generation, handling and integration

• model building and testing followed by refinement.

We also exploit the models to find new insights into biological mechanism and human disease, and to develop approaches for ciliotherapies.

http://syscilia.org/

